# Group Music Training and Children's Prosocial Skills

**DOI:** 10.1371/journal.pone.0141449

**Published:** 2015-10-27

**Authors:** E. Glenn Schellenberg, Kathleen A. Corrigall, Sebastian P. Dys, Tina Malti

**Affiliations:** Department of Psychology, University of Toronto Mississauga, Mississauga, ON, Canada; UNLV, UNITED STATES

## Abstract

We investigated if group music training in childhood is associated with prosocial skills. Children in 3^rd^ or 4^th^ grade who attended 10 months of music lessons taught in groups were compared to a control group of children matched for socio-economic status. All children were administered tests of prosocial skills near the beginning and end of the 10-month period. Compared to the control group, children in the music group had larger increases in sympathy and prosocial behavior, but this effect was limited to children who had poor prosocial skills before the lessons began. The effect was evident even when the lessons were compulsory, which minimized the role of self-selection. The results suggest that group music training facilitates the development of prosocial skills.

## Introduction

Throughout history, some form of music has been evident in all human cultures [1–3]. Evolutionary theorists posit that music may be an adaptation because it facilitates social cohesion [4–7]. Tightly knit social groups with greater levels of sympathy, helping behaviors, altruism, and so on, are thought to have an increased likelihood of warding off threats from other groups, predatory animals, and the environment. One might expect, then, that music making enhances children’s cooperative, prosocial behaviors, such as voluntary actions that benefit others [8].

The available developmental research has focused almost exclusively on associations between music lessons and *cognitive* abilities. Music training in childhood has positive associations with abilities in a variety of domains, including speech perception, other language abilities (i.e., reading, vocabulary, spelling, second-language acquisition), and visuo-spatial abilities, as well as with domain-general abilities such as memory, executive functioning, intelligence, and academic achievement [9]. Cognitive abilities also tend to increase in tandem with duration of music lessons [10,11], and there is some evidence that music training *causes* small enhancements in these areas [12–14]. Associations in real-world studies [15–18] tend to be much larger than those in experimental studies, however, which could be a consequence of longer-duration training. Pre-existing individual differences in cognitive ability, personality, and demographic background are also likely to be implicated [10,19].

When developmental researchers ask whether music training is associated with social and emotional skills, null or inconsistent findings often emerge, although these could be a consequence of the measures that are used. For example, parents' reports of their children's cooperative behaviors are *not* associated with the child's music training [11,14]. Even when children are asked for self-reports about their levels of self-esteem [20–22], effects are weak or nonexistent. For example, in a three-year study of children assigned to piano lessons or no lessons, improvements in self-esteem were evident for the piano group but not for the control group [20]. Nevertheless, change over time in self-esteem did not differ significantly between groups (i.e., no group X time interaction), and the two groups did not differ at any time they were tested. In a two-year study that reported effects of music training on cognitive ability, music training had no effect on children's perception of their social status [13]. Another study found no effects of 6 months of school-based music training on 10- to 13-year-olds' reports of self-esteem [21]. In a sample of younger children, however, a year of school-based music training tempered the declines in self-esteem that were evident among controls [23]. Finally, although musically trained 7- and 8-year-olds show enhanced performance on a test of emotion comprehension, this association is due to their relatively high IQs [18]. In short, it is impossible at present to reach any definitive conclusions about music training and social skills.

Studies with null findings have often examined individual music lessons with one student and one teacher [11,20], or group lessons with minimal interaction among students [14]. Lessons taught in groups, especially those that promote positive interactions and cooperation among students, may reveal positive findings [24]. In line with this view, drama lessons—which are interactive by nature—improve social skills (e.g., cooperating with adults, empathy, theory of mind), whereas music lessons with minimal interaction among students do not [14,25]. Positive findings are also evident in interventions that combine drama with dance in order to facilitate children’s prosocial skills and self-esteem [26]. Among adults, attending music concerts—a group activity—is associated positively with prosocial behaviors such as voting, donating to charities, volunteering, and community involvement, even after holding constant potential confounding variables such as age, sex, race, income, education, marital status, and occupational status [27].

Suggestions that group music making *causes* improved social and emotional skills come primarily from programs that were designed specifically to promote such skills. In one instance [28], random assignment of 8- to 11-year-olds to 9 months of interactive music-making in groups of four to eight children led to increases in empathy. The music group also exhibited an advantage in remembering the emotions conveyed by photographs of facial expressions. Although the program was successful, it involved a “specially tailored” curriculum of musical games that differed markedly from standard music training.

Another study examined joint music-making among 4-year-olds [29]. It involved two brief sessions of pretend play in the laboratory with the experimenter and one other child. The sessions included a repetitive playsong for some children but no music for other children. The condition with the playsong led to a greater incidence of helping (i.e., when one of the children “accidentally” spilled her set of marbles) and increased levels of cooperation in a problem-solving task.

In a study of 6-month-olds [30], researchers compared programs of active music engagement and passive music listening. The “active” condition comprised weekly interactions with a parent and two music teachers, which included singing and moving to music. The “passive” condition involved play sessions while music was presented in the background. Infants in the active condition showed larger increases on parent-report measures of positive affect (smiling and laughing) and soothability, and larger decreases on measures of distress and wariness of novelty. All of these studies lacked ecological validity, however, in the sense that they were conducted in the laboratory [29], they involved nonstandardized programs that were designed specifically to improve social skills [28], or they involved children who were much too young for typical instrumental or vocal music lessons [30]. In other words, it is unclear whether the findings would generalize to school-age children’s experiences with music in everyday life.

The present study was a natural experiment that examined whether social benefits are accrued from more typical group music training, specifically an existing program that was designed with music pedagogy as its focus. The participants were 8- and 9-year-old children, the age at which the program was designed to be initiated. Some of the children attended public schools that incorporated this specialized program, which included learning to play the ukulele, singing, and much interaction among students. Other children attended public schools without such a program. All children were pretested in the fall of the school year (Time 1, hereafter T1) to provide baseline measures on four outcome measures: vocabulary, emotion comprehension, sympathy, and general prosocial skills. The children were retested at the end of the school year (Time 2, hereafter T2) to determine whether any change from T1 to T2 might differ between groups. For the majority of the children in the music program (74%), participation was mandatory, which minimized the role of self-selection.

We measured sympathy rather than empathy because sympathy entails other-oriented concern, whereas empathy involves the experience of the same or a similar emotion as someone else [31]. Because of its orientation toward another person, sympathy is thought to be an important motive of prosocial behavior [8]. We predicted that the music lessons would be accompanied by improvements in sympathy and prosocial behaviors, but not in vocabulary. Vocabulary is a marker of general cognitive ability [32], and to date there is little evidence that group music lessons cause improvements in cognitive abilities [9,33]. We had no prediction about emotion comprehension, because even though it is part of social skills, abilities on the test we used are highly correlated with language skills and general cognitive abilities [18,34,35]. Thus, we expected group differences after the music lessons on some measures but not on others. Because intervention programs can have the greatest success among children who need them the most [36,37], we also expected that group music making would be most beneficial for children who began the program with poor social skills.

## Materials and Methods

### Ethics Statement

Signed written consent was obtained for all participants from a parent or legal guardian, and the children assented to participate orally. The study protocol was approved by the Research Ethics Board of the University of Toronto and the Toronto District School Board. The methods were carried out in accordance with guidelines provided by the Tri-Council policy statement on ethical conduct for research involving humans.

### Participants

The participants were 84 3^rd^ (*n* = 56) and 4^th^ (*n* = 28) graders enrolled in public schools in a major Canadian city. The sample comprised 49 girls and 35 boys, who were, on average, 8 years and 8 months at T1 (*SD* = 7 months). English was the native language for 90% of children. When asked about number of siblings, the modal response was 1 (56%); 15% had no siblings and 29% had 2 or more. Approximately one-third of mothers and fathers were university graduates, with the remaining two-thirds divided roughly equally into groups with less or more education. Yearly family income varied widely. Approximately half of the children came from homes with incomes over $125,000. Although the modal response (19%) was in the $125000-$150000 range, 17% (the next most common response) were in the $50,000-$75,000 range.

Children in the music group (*n* = 38) attended schools that incorporated an enhanced group music program into the curriculum. The lessons were mandatory for all 3^rd^ graders at one school (*n* = 28), but optional for 4^th^ graders at another school (*n* = 10). Children in the control group (*n* = 46) attended schools in neighborhoods with similar socio-economic status (SES) but without the enhanced music program.

### Music Program

The music classes were weekly 40-min sessions held in a classroom over the course of the school year (10 months). The music program was developed in the 1970s by J. Chalmers Doane, who was director of music education for a school board from 1967 to 1984. His goal was to make primary-school children musically competent by the end of 6^th^ grade. The most innovative and distinguishing feature of his program is the use of the ukulele in classroom music lessons. The ukulele is an affordable and child-friendly instrument that can be played alone or in groups. Ukuleles have nylon strings that are less harsh than steel strings on children’s fingers, and the four strings are tuned in the same intervals as the four highest strings on the guitar, which means that much of what is learned in terms of fingering can be transferred to guitar.

The establishment of the non-profit *Ukulele in the Classroom* program (www.ukuleleintheclassrooom.com) in 2008 was a collaboration between Doane and James Hill. The program incorporates the use of hand signs (as in the Kodály method), ear training, musical notation (treble clef), sight reading, pentatonic and diatonic scales, duple and triple meters, solo and ensemble performances, improvisation, and much singing. The repertoire includes arrangements of music taken from classical, jazz, traditional /folk, world, and popular genres. The teaching philosophy is one of “teaching to the top.” Children who learn readily are encouraged to “show your neighbor”—to pass on what they know to other students. The children are told specifically that it is *not* cheating to look at or help their neighbor, and that musicians use their eyes and ears to cue each other about what is coming next in a performance. This pedagogical approach is part of a broader strategy to encourage co-operation and positive interactions during the process of learning to make music.

### Measures

#### Vocabulary

Vocabulary was measured with the Peabody Picture Vocabulary Test—Fourth Edition [32], a well-known measure of receptive vocabulary. On each trial, children saw four drawings while the examiner said a word. The child’s task was to point to the picture that corresponded to the meaning of the word. The test provides standardized scores based on age such that a score of 100 (*SD* = 15) is the average for the child’s age group in the US general population.

#### Emotion comprehension

We administered seven of the nine subtests from the Test of Emotion Comprehension [38], omitting the first two subscales, which are designed for younger children. On each trial, the examiner read a brief scenario and the child was asked to identify the emotional state of a same-sex protagonist by pointing to one of four illustrations of facial expressions. The outcome variable was the aggregate score formed from performance on the seven subtests. Scores ranged in principle from 0 to 7.

#### Sympathy

Sympathy was measured with the six-item Child-Report Sympathy Scale [39], modified slightly so that children made responses on a 6-point instead of a 3-point scale (1 = not at all like me; 6 = very much like me). Items were read aloud to children (e.g., *When I see another child who is hurt or upset*, *I feel sorry for them*. *Does this sound like you*?), who responded by pointing to one of the six options. An additional item with similar content (*Do you feel sorry for another kid who has made a mistake*?) was included to improve internal reliability. Scores were averages of the seven ratings. Cronbach’s α for the 7-item sympathy scale was .67 at T1 and .65 at T2.

#### Prosocial skills

Overt prosocial behavior was measured with the 10 prosocial items from the Social Behavior Questionnaire [40], modified for self- instead of teacher-report. Items assessed helping (e.g., *If a kid has been hurt*, *will you try to help like Tom does*?), sharing (e.g., *Do you share your things with others*, *as Tina does*?), conflict resolution (*If there is an argument between children*, *do you try to stop it*?), and so on. For each item, a colored drawing of the protagonist (Tom for boys, Tina for girls) committing a prosocial act was shown to the child while the examiner asked the question orally. The 6-point response scale was the same as the one used to measure sympathy. Scores were averages of the 10 ratings. Cronbach’s α was .76 at T1 and .81 at T2.

### Procedure

Testing occurred in the fall of 2012 and the late spring and summer of 2013. All measures were administered on both occasions. Children were tested individually in a quiet room. The parent, if present, completed a questionnaire used previously [10,11,17–19]. Otherwise, the questionnaire was sent home with the child. It asked for demographic information, including annual family income, which was measured on a 9-point scale in increments of $25,000 (1 = below $25,000, 9 = above $200,000), and parents’ education, which was measured on an 8-point scale (1 = did not finish high school, 8 = graduate degree) separately for both parents. It also asked for details about the child’s native language and number of siblings, and for subjective ratings (on 7-point scales) about the child’s musicality: (1) *How musical is your child compared to the average child*? and (2) *How interested is your child in music compared to the average child*?

## Results

Preliminary analyses confirmed that the music and control children were similar at TI in terms of mother’s education, *p* > .1, father’s education, *p* > .1, family income, *p* > .4, gender composition, *p* > .4, age, *p* = .095, number of siblings, *p* > .1, English as a first language, *p* > .7, and parent’s ratings of the child’s musicality, *p* > .5, and interest in music, *p* > .2.

As one would expect, correlations between scores at T1 and scores at T2 were significant for each outcome variable: vocabulary, *r* = .803, emotion comprehension, *r* = .414, sympathy, *r* = .511, and prosocial skills, *r* = .660, *N*s = 84, *p*s < .001. For each test, children who performed well or poorly at T1 also performed well or poorly, respectively, at T2. Table 1 provides correlations among the different outcome measures separately for T1 and T2. Both matrices are corrected for multiple (i.e., 6) tests. At both time points, vocabulary had a positive association with emotion comprehension, and sympathy was correlated positively with prosocial skills and emotion comprehension. Vocabulary was associated positively with sympathy at T1 but not at T2.

**Table 1:** Correlations Among Outcome Variables and Time 1 and Time 2 (*N* = 84).

**TIME 1**			
	**Vocabulary**	**Emotion Comprehension**	**Sympathy**
**Emotion Comprehension**	.334*		
**Sympathy**	.295*	.418*	
**Prosocial Skills**	.083	.229	.509*
**TIME 2**			
	**Vocabulary**	**Emotion Comprehension**	**Sympathy**
**Emotion Comprehension**	.328*		
**Sympathy**	.037	.237*	
**Prosocial Skills**	.138	.210	.766*

* *p* < .008

The main analyses examined whether the difference between T1 and T2 varied as a function of group (music or control) and initial levels of performance (low or high), separately for each outcome measure. For each outcome measure, a median split was used to divide the children into low or high scorers at T1. Because of tied scores, the low and high scorers never had exactly equal numbers (i.e., the largest imbalance was 39 v. 45). Mixed-design Analyses of Variance (ANOVA) examined scores as a function of one repeated measure (testing session) and two between-subject factors (group, initial performance). Thus, each participant was in one of four cells based on group and initial performance, and each participant had two scores (T1 and T2) for each outcome measure. Descriptive statistics are provided in Table 2.

**Table 2:** Means (and SDs) at Time 1 and Time 2.

	**Low Scores at Time 1**							
	**Music Group**				**Control Group**			
**Measure**	**Time 1**		**Time 2**		**Time 1**		**Time 2**	
**Vocabulary**	103.16	(8.15)	105.32	(12.83)	105.46	(7.24)	105.96	(12.51)
**Emotion Comprehension**	4.29	(0.61)	5.55	(1.08)	4.51	(0.53)	5.49	(1.20)
**Sympathy**	4.32	(0.70)	5.09	(0.74)	4.41	(0.70)	4.59	(0.59)
**Prosocial Skill**	4.62	(0.39)	5.14	(0.49)	4.53	(0.46)	4.57	(0.54)
	**High Scores at Time 1**							
	**Music Group**				**Control Group**			
**Measure**	**Time 1**		**Time 2**		**Time 1**		**Time 2**	
**Vocabulary**	127.68	(12.87)	126.26	(13.85)	123.45	(7.49)	117.80	(10.28)
**Emotion Comprehension**	6.29	(0.52)	6.20	(0.59)	6.25	(0.53)	6.04	(0.81)
**Sympathy**	5.64	(0.25)	5.47	(0.36)	5.65	(0.21)	5.55	(0.45)
**Prosocial Skill**	5.48	(0.30)	5.23	(0.46)	5.56	(0.23)	5.46	(0.45)

For vocabulary, there was a two-way interaction between testing session and initial performance, *F*(1, 80) = 6.32, *p* = .014, partial η^2^ = .073. For children who scored below the median at T1, there was no change over time, *F* < 1. Recall that PPVT scores are standardized based on age, so one would expect little change as a simple consequence of age. For high scorers, however, there was a *decrease* from T1 (*M* = 125.51, *SD* = 10.54) to T2 (*M* = 121.92, *SD* = 12.73), *F*(1, 37) = 8.00, *p* = .008, partial η^2^ = .178. In any event, change over time was unrelated to group assignment, *p*s > .1 (i.e., no two-way interaction between testing session and group, no three-way interaction).

For emotion comprehension, there was a similar but stronger two-way interaction between testing time and initial performance, *F*(1, 80) = 38.88, *p* < .001, partial η^2^ = .327. Children who scored low at T1 showed marked improvement from T1 (*M* = 4.41, *SD* = 0.57) to T2 (*M* = 5.52, *SD* = 1.13), *F*(1, 38) = 45.61, *p* < .001, partial η^2^ = .546. High scorers showed no change, *p* > .2. As with vocabulary, change over time was unrelated to group assignment, *p*s > .3.

**Fig 1:**
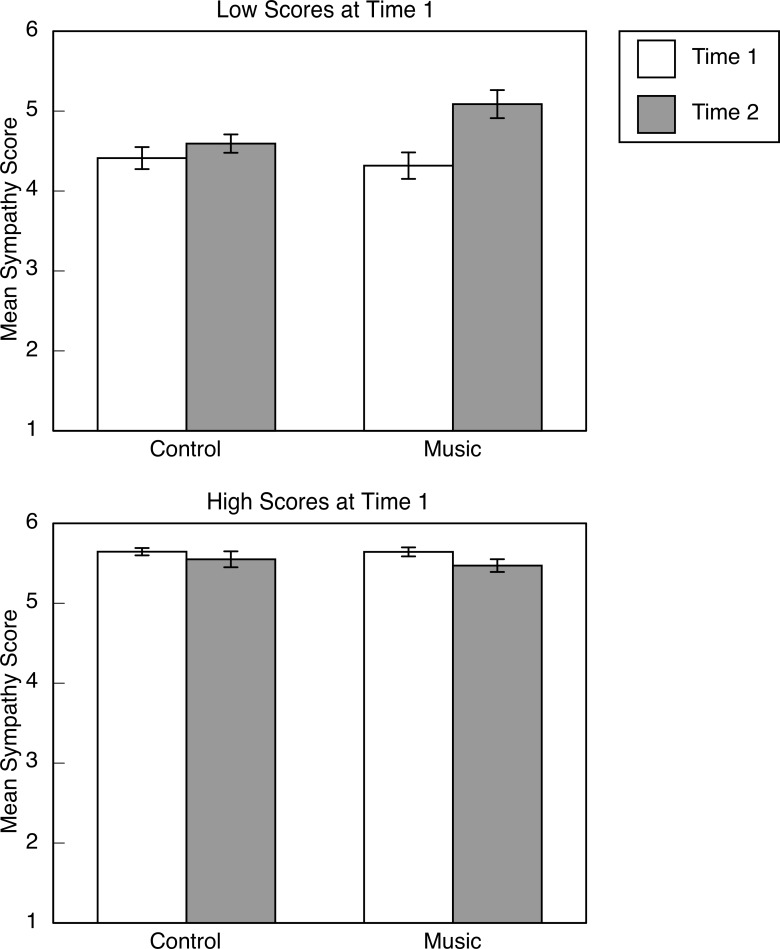
Group music training was associated with increases in sympathy scores. Mean sympathy scores as a function of testing session and group assignment. Scores from children who scored below and above the median are illustrated in the upper and lower panels, respectively. Error bars are standard errors.

The results from the ANOVA on sympathy scores revealed a three-way interaction that was in line with our predictions, *F*(1, 80) = 4.94, *p* = .029, partial η^2^ = .058. Descriptive statistics are illustrated in Fig 1. The interaction motivated separate examination of children who scored in the higher or lower half at T1. For high scorers, there was no main effect of group, *p* > .6, and no interaction between group and test session, *p* >.5. There was, however, a main effect of test session, *F*(1, 38) = 4.26, *p* = .046, partial η^2^ = .101. Regardless of group assignment, children with high sympathy scores at T1 (*M* = 5.64, *SD* = 0.23) tended to have slightly lower scores at T2 (*M* = 5.51, *SD* = 0.40). For children who scored in the lower half at T1, a different pattern emerged. There was a significant interaction between group and test session, *F*(1, 41) = 5.07, *p* = .030, partial η^2^ = .108. Children in the music group showed increases in sympathy from T1 to T2, *t*(17) = 3.45, *p* = .003, but children in the control group did not, *p* > .2. These results were similar when the 10 children who chose to take the ukulele classes were excluded from the analyses. The only difference was that for children who scored in the upper half at T1, the reduction in sympathy from T1 to T2 was only marginally significant, *p* = .091. For children who had low scores at T1, nothing changed.

**Fig 2:**
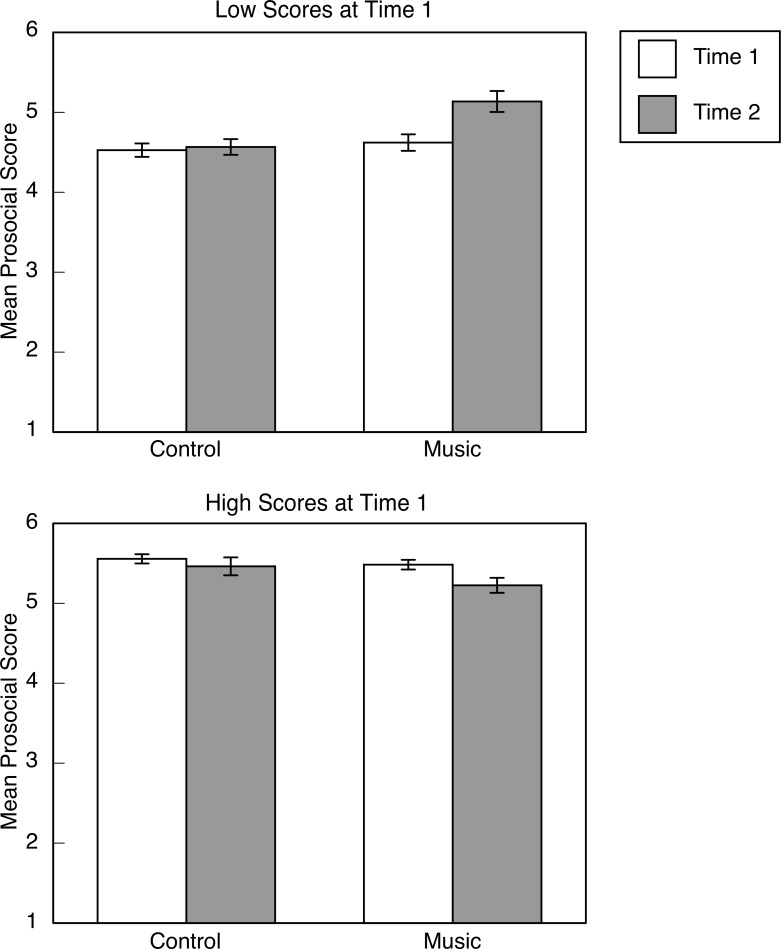
Group music training was associated with increases in prosocial scores. Mean prosocial scores as a function of testing session and group assignment. Scores from children who scored below and above the median are illustrated in the upper and lower panels, respectively. Error bars are standard errors.

The analysis of prosocial skills also revealed a three-way interaction, *F*(1, 80) = 10.45, *p* = .002, partial η^2^ = .116, which is illustrated in Fig 2. Follow-up tests involved separate examination of children who scored above or below the median at T1. For children who scored in the upper half at T1, there was a main effect of test session, *F*(1, 38) = 8.72, *p* = .005, partial η^2^ = .187, but no main effect of group, *p* > .1, and no two-way interaction between group and test session, *p* > .1. Regardless of group assignment, children who scored high at T1 (*M* = 5.51, *SD* = 0.27) tended to score lower at T2 (*M* = 5.32, *SD* = 0.46). For children who scored in the lower half at T1, there was a two-way interaction between group and test session, *F*(1, 42) = 9.23, *p* = .004, partial η^2^ = .180. Children in the music group improved from T1 to T2, *t*(13) = 3.49, *p* = .008, but children in the control group did not, *p* > .6. Removal of the 10 children who voluntarily took the music lessons had no effect on the results.

Although the two groups of children did not differ on any demographic variable, it is still possible that small but nonsignificant differences between groups on one or more of these variables could have contributed to the group differences in social skills reported above. To test this possibility, we added covariates and re-ran the initial ANOVAs for sympathy and general prosocial skills. We first added age (measured in days) because age was the only variable that showed even a marginal difference between groups. The crucial three-way interaction remained significant for sympathy, *p* = .044, and for prosocial skills, *p* = .002. We also held constant *all* demographic variables (i.e., mother’s education, father’s education, family income, gender, age, number of siblings, English as a first language, and parent’s ratings of the child’s musicality and interest in music). The results did not change. For sympathy, *p* = .021, and for general prosocial skills, *p* = .023, the three-way interaction remained significant. We also used discriminant-functions analysis to confirm that no linear combination of the demographic variables could reliably determine group membership, *p* > .1. In short, the significant effects we observed could not be attributed to between-group differences in demographics.

## Discussion

Group music training and prosocial skills were associated positively among 8- and 9-year-olds, but only for children who had poor social skills at the beginning of the study. Unlike most developmental studies of associations between music training and nonmusical abilities, self-selection played a minimal role because the group lessons were compulsory for most children, and the findings did not change when children who voluntarily took the lessons were excluded from the analyses. In contrast to programs designed for infants [30], and those designed specifically to improve social behavior [22,28], music training in the present investigation involved an existing music program designed for children in primary school. These findings are consistent with the view that music is an adaptive behavior because it fosters social cohesion, cooperation, and a prosocial orientation.

Although results from natural experiments do not lend themselves readily to inferences of causation, we can be certain that better social skills at T2 did not cause children to take group music lessons beforehand. Demand characteristics are also likely to have been minimal because the children were unaware of their scores at T1 and the study’s hypotheses. Although we cannot rule out the possibility that an unknown variable caused some of the improvements in social skills that were evident for the children in the music program, it seems likely that the program played a substantial role. It remains unknown, however, whether nonmusical group activities would have a similar effect on prosocial skills [14]. For example, other activities that emphasize group interaction and cooperation, such as drama lessons, could lead to similar benefits. It is also possible that experimenter expectancies played a role, with subtle but unintentional cues leading children to respond in desired ways. Unfortunately, the music program was implemented at some schools but not others, which made it impossible for the experimenters to be blind to group assignment. Nevertheless, the experimenters were unaware of children's performance at T1. Because some children scored lower at T2 than they did at T1, whereas others scored higher, it seems unlikely that experimenter bias was driving response patterns.

We did not find any effect of music training for children who began the program with elevated levels of social skills. For children who scored high at T1, there may have been little to no room for improvement on our measures of sympathy or general prosocial skills. Indeed, average scores for these children were above 5 on our 6-point scales (see Table 2). In some instances, children who scored high at T1 actually showed lower levels of performance at T2. This was true for sympathy and general prosocial skills, as well as for vocabulary. Regression to the mean was likely to be involved, or, perhaps, reductions in motivation among high-performing children when they took the same test a second time. One might wonder whether regression to the mean could also account for improvements that were evident among children who scored low at T1. This is a possibility, at least in principle, but there is no way to explain why it would be more of a problem for children in the music group than for children in the control group.

What underlying mechanisms would lead group music training to enhance prosocial skills? We consider two possibilities: peer interaction in general, and synchrony among peers in particular [24]. Children’s peer relations provide a valuable context for their social and emotional development [41,42]. Vygotsky highlighted the role of collaboration between children in promoting shared understanding and co-construction of knowledge and skills [43]. More specifically, he emphasized the importance of guided learning for tasks that children cannot accomplish on their own, but are able to complete with the help of a more knowledgeable child or adult. In the present study, children could easily hear if they or their peers were struggling with a lesson (e.g., playing or singing wrong notes or the right notes at the wrong time), and the music program stressed that children should help those who were having difficulty.

Children’s intrinsic interest in music is also likely to foster cooperation, particularly in the context of a social environment that emphasizes social-emotional development over academic factors such as grades in school [44]. Such collaboration may improve children’s social bonds, and increase their prosociality by raising their motivation to provide support for others, and their willingness to receive help from others. Helping behaviors (i.e., giving and receiving) are known to foster a sense of security among friends and peers [45–47].

Music lessons taught in groups also offer a social context for the development of *synchrony* [48]—the temporal coordination of biological events, social behaviors, or affective states. Music is an ideal candidate for facilitating synchrony because it provides an external rhythmic framework that facilitates coordination among individuals [7]. In our group music classes, motor movements were synchronous, often simultaneously, for three different body parts: the mouth (singing), right hand (strumming the ukulele), and left hand (finger positions on the ukulele). Correlational studies have linked synchrony during parent-child interactions to children’s mental health, moral-emotional development, and positive behavioral outcomes [49]. These findings generalize to experimental paradigms, studies of peers in childhood, and adult studies: Activities that encourage high levels of motor synchrony (e.g., dancing, singing, finger-tapping) increase cooperative, other-oriented behaviors and/or well-being in children and adults [29,50–55].

Synchrony can lead to a release of endorphins [56,57], which are important for facilitating non-kin, non-sexual social bonds [58,59]. Synchrony also appears to facilitate the sharing of emotional experiences [60], and to promote self-other merging, both of which are central to the development of other-oriented emotions [61]. Even in the laboratory, group synchrony emerges spontaneously in terms of movements and breathing patterns [62]. More generally, the inherently social and communicative nature of music would make group training an excellent vehicle for increasing the coordination of behavior, affect, and mental states among children [48,51,53,63]. Over time, children’s increased connectedness to a world of mental states shared with others could give rise to other-oriented emotions, such as sympathy, and promote other-oriented behaviors [64–66]. In short, the synchrony that was involved in singing and playing the ukulele in groups was likely to have played a major role in prosocial development, and in the differences between the treatment and control groups that we observed.

It remains unclear whether the effects we observed here would last for months or years after the group music lessons, whether training of longer duration would be associated with even better social skills, and whether all children might show benefit if more sensitive measures were used. Nonetheless, the present findings suggest that (1) group music lessons are a contributor to social cohesion, by increasing children’s sympathy and prosocial behavior, and (2) these effects are evident only among children who begin the lessons with poor social skills. Sympathy is likely to be a motivational force that spurs prosocial behavior [8,67], which leads to concomitant reductions in antisocial behavior such as bullying and aggression [68]. More generally, increases in prosocial behavior coupled with decreases in antisocial behavior strengthen group trust and security [45–47] and further promote children’s healthy behavioral functioning. In any event, our findings provide a clear hypothesis that could be tested in future interventions with at-risk children.
